# Referral of sick children and levels of adherence by carers: implications on quality health care in the middle belt of Ghana

**DOI:** 10.4314/gmj.v55i1.7

**Published:** 2021-03

**Authors:** Mathilda Tivura, Samuel Afari-Asiedu, Martin Adjuik, Frank Baiden, Seth Owusu-Agyei

**Affiliations:** 1 Kintampo Health Research Centre, Ghana Health Service, Kintampo, Ghana; 2 School Of Public Health. University of Health and Allied Sciences, Ho, Ghana; 3 Institute of Health Research. University of Health and Allied Sciences, Ho, Ghana

**Keywords:** Referral, Adherence, health care system, developing countries, central Ghana

## Abstract

**Introduction:**

Severely sick-children presenting at primary healthcare facilities need referral to higher level facilities for better care. Adherence to referrals and quality of care received by those referred could serve as critical steps towards their survival.

**Objective:**

To describe experiences with severely sick children referred to higher-level health facilities for care and reasons for non-adherence to referral; to explore healthcare provider's perspectives to referral.

**Methods:**

Referrals among 3046 young children were followed for adherence. Assessment of children referred from a PHC facility adhering to referral advice and reasons for non-adherence to referral was determined. Agreement on reported diagnoses at PHC centres and health-facilities receiving patients was assessed. Perspectives of healthcare providers were assessed.

**Results:**

212 children were referred from PHC centres to various hospitals with 14.2% non-adherence. Reasons given: 48.3% of carers adhering felt child's condition was severe; 43.3% complied with healthcare provider directive. The main reasons for non-adherence to referral were no money for transport (50%) and child condition not serious (30.0%). 69.0% of anaemia cases diagnosed at PHC facilities and hospitals. 65.7% fever diagnosed at a PHC centres were confirmed as malaria at the hospitals. Healthcare providers referred patients for severity, perceivedcomplication and non-response to treatment.

**Conclusion:**

Adherence was generally good. The level of agreement in diagnosis of common diseases such as malaria and anaemia at PHC centres and district hospitals was high and low for rarer diseases. Capacity should be provided at PHC levels for adequate management of cases presented to reduce referrals carers have to make

**Funding:**

This study did not receive funding from any external sources

## Introduction

Approximately 6.5 million children in developing countries die every year before reaching their fifth birthday, with a significant proportion of deaths occurring during the first year of life.^1^, ^2^ More than half of these deaths are due to preventable or easily-treatable diseases.^3^, ^4^ Majority of these deaths could be prevented at Primary Healthcare (PHC) facilities that appropriately treat or refer patients to next level of care.^5^

Whilst every ill-child require prompt, appropriate medical attention,^2^,^6^–^8^ most PHC facilities are constrained in managing illnesses.^9^–^11^ Presenting illness in resource-limited and under-capacity PHC facilities for management require children being referred to higher-level facilities.

Some carers of such children do not always comply with referrals^11^,^12^ Examples of limited resources include lack of laboratory buildings, laboratory equipment and supplies and under-capacity include lack of qualified personnel.

In Ghana, PHC facilities comprise of health centres, community-based planning services (CHPS) and represents the first level of institutional care, which offers proximity and uses relatively low-level technology. District hospitals represent the secondary level that take care of patients referred from PHC level and regional/teaching hospital represents tertiary level.^4^,^5^ PHC facilities serve predominantly rural populations, and are manage by low to middle-level health personnel, comprising community health nurses, general nurses, midwifes and physician assistants.^13^ These facilities lack clinical laboratories and therefore resort to presumptive clinical management.

Severely ill children seen at PHC are referred to higher level health systems for better care. It has been documented in other reported studies that not all carers whose children are referred from the PHCs comply by taking their sick children to the higher level facility.^4^, ^14^ Caregivers often expect that their children will be cured at their first contact with healthcare providers.^15^, ^16^ When this expectation is not met and they are referred to the higher level, some carers are naturally disappointed. A carer who is not able to meet costs at a higher level facility usually seek alternative solutions^4^, ^17^, with defaulting in referral resulting in detrimental health outcomes.

A randomised-control trial involving 3046 children aged two to 24 months in five-adjoining districts in the Brong-Ahafo Region of Ghana was conducted between 2010 and 2012. It compared effect of test-based management and presumptive management of malaria among patients who presented at the PHC facilities or referred to the PHC facilities following monthly visits to their homes over a 24-months period.^9^

We observed from the main study^9^ that not all carers whose children were referred adhered and took the children to the higher-level facilities. We therefore incorporated a sub-study to describe our experiences with referrals during the study. Diagnoses at primary and higher level facilities, health worker perspectives of the referral process and adherence to referral advice with reasons for non-adherence were explored. In our study, we did not directly work with the second and third level facilities. The Management and Staff of these hospitals, however, provided us with information of the patients involved.

## Methods

### Study setting, design and sampling procedures

Study setting, design and procedures in the Randomised Controlled trial (RCT) were reported elsewhere.^9^ The RCT involved a cohort of 3046 young children residing within 2km of 32 primary healthcare centres, that were randomised in equal halves to provide either test-based management (the intervention arm), or presumptive management (the control arm), for malaria whenever they presented with fever or suspected malaria.

Access to PHC and district hospitals was not different for children who participated in the study. The guidelines contained in the Integrated Management of Childhood Illness (IMCI) approach^14^,^18^ was used in all 32 health facilities to standardise clinical management of cases of fever in RCT children.

Rapid diagnostic test (RDT) for malaria was used for participants in PHC randomised to test-based approach while clinical judgement was used in PHC randomised to presumptive approach.

### Ethical approval and consent to participate

Ethical approval (Ref: khrc/iec/FEA/2008-37) was obtained from the Kintampo Health Research Centre Institutional Ethics Committee FWA (KHRC-IEC) with FWA registration 00011103. Written informed consent was obtained from all study respondents. Participants/respondents were informed of the voluntariness of their participation. They were also assured of confidentiality of the information they provided.

### Design of referral study and sampling procedures

All study children seen by providers in the PHC facilities requiring referral, based on IMCI guidelines, were referred to nearest district/municipal hospital located within the study area.

A research assistant (RA) stationed at each of the 32 PHC facilities documented all study participants visits and referrals to district hospitals. Basic data including, facility referring and referred to and reason(s) for referral were recorded and shared regularly with the study clinical coordinator to aid follow-up. Besides being stationed at PHCs, RAs visited each study participant at home once a month to check on their health status and whether they have visited any health facility in the intervening period.

### Follow-up survey

Between June 2012 and September 2012, we interviewed all parents/carers whose children were referred (n=212) using structured questionnaire that explored reasons for adherence and non-adherence to referral. We used the information we have obtained through monthly visits to help parents/carers to recall events during the main trial. They were asked why they decided on what they did about the referral. They were also asked about the child's current status, reason for referral, facility to which the child was referred, whether carer adhered to referral, reasons for adherence or non-adherence. For those who did not adhere, any care given at home was documented, whilst treatment given at district health facility for those adhering was documented as well.

### Diagnoses at PHC and referral hospital

At PHC, sick children were seen at outpatient departments and depending on child's condition, attending health worker requests laboratory tests followed by treatment or manages child on presumptive treatment. Those identified with severe diseases were referred from PHC to district hospitals where they are further examined and managed based on results.

Agreement on reported diagnoses at PHC referring the cases and the referral facilities that received patients was assessed amongst carers adhering to referral (n=182).

### Follow-up in-depth interviews

In-depth interviews (IDIs) were conducted with health workers who made referrals to obtain their perspectives on reasons for carer adherence and non-adherence to referral advice. A total of 10 IDIs were conducted in 10 purposively selected PHC centres, one IDI per selected PHC centre. Each IDI lasting about 30 to 45 minutes was facilitated by a moderator and a note-taker, who explored themes on referrals, including reasons for referral, challenges with referrals and reasons for accepting or refusing referrals. Interviews were digitally recorded and transcribed verbatim soon after each interview. Interviews conducted in the local language (Twi) were translated into English language before transcription.

### Data management and analysis

Completed structured questionnaires were checked for consistency and double-entered in MS Access. The data was exported to Stata 12 for analysis. For descriptive analysis frequencies and cross-tabulations of categorical variables were run. For continuous variables the means and standard deviations were reported. This was followed by an analysis, comparing independent diagnoses made at PHC and hospital levels and the level of agreement between the two sets of diagnoses was assessed using simple proportions. Response categories of “Other” and ‘No diagnosis” were dropped leaving 171 records, which cross-tabulations were conducted.

Transcripts from qualitative interviews were imported into NVivo 8 and coded according to thematic areas generated from the survey. Prior to coding, 1(10%) of the transcripts were reviewed and coded independently by two persons to ensure inter-coder agreement. This was followed by a debriefing session to discuss level of consensus. In the process of coding, additional themes were included inductively to the coding framework as determined by the data.

## Results

The cohort of 3046 children had a total of 12,873 contacts with the 32 PHC centres identified during the period of follow-up, suggesting that on average, each child was taken to a PHC 4–5 times during the period of the study.

### Adherence to referral from PHC to hospital

**Figure 1:**
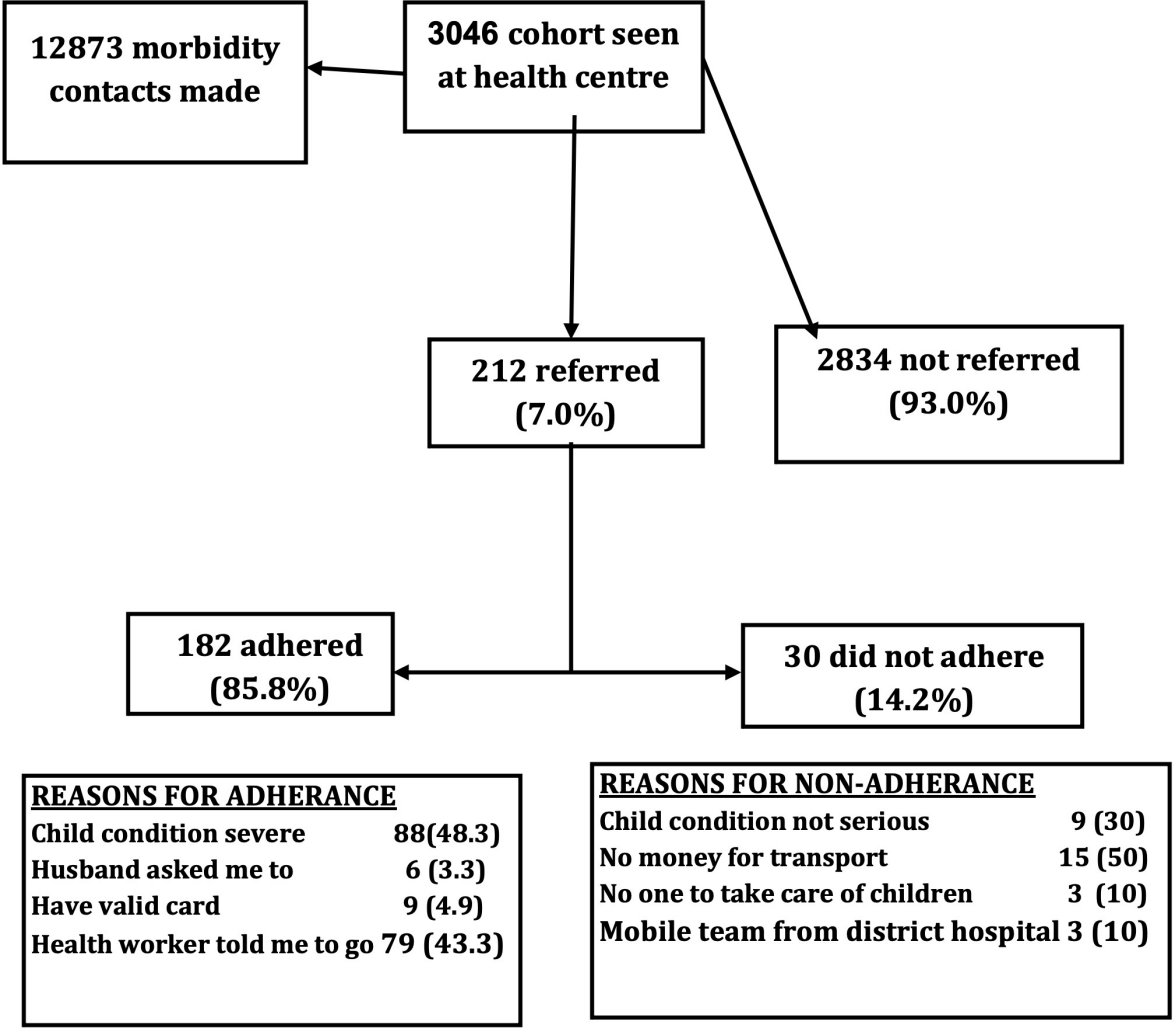
Study Profile

A total of 212 (≈7.0%) children were referred from a PHC to a hospital located within the study area (Figure 1). The remaining 2834 (93.0%) were seen at PHC centres but not referred. Carers of 30 (≈14.2%) of those referred to a hospital did not adhere to referral.

The reasons given by carers who complied with referral were: felt child's condition was severe enough requiring referral to a higher level, complying to health workers instruction to send the child to a higher level of care, husband insisting on wife sending child to hospital and possession of valid national health insurance. Reasons for non-compliance were mother felt child's condition was not that serious requiring referral, no money for transport, no one to take care of other siblings and expected mobile team into the community that will take care of the child (Figure 1).

### Diagnoses at Primary and Referral Facilities

Diagnoses made at PHC level included fever, malaria, anaemia, cough, diarrhoea, constipation, abdominal pains, vomiting, skin disease, skin infection, URTI among others and most of these were diagnosed presumptively, using IMCI. In the hospitals, there were overlapping diagnosis with those made at PHC centres, but the hospitals were able to make further diagnosis backed by laboratory and clinical support. Reports of diagnoses such as confirmed malaria, anaemia, hepatitis, pneumonia, sepsis, asthma, gastroenteritis were made in the hospitals.

The main diagnoses at PHC centres were anaemia (68), malaria (50), fever (45), convulsion (13) and diarrhoea (8). Diagnoses such as: “Pneumonia”, “Asthma”, “Constipation”, “Cough”, “Dislocation”, “Oral thrust”, “Restlessness”, “Skin disease”, “Typhoid”, “Weakness”, “Yellow coloration” and “Yellow fever”. were assigned to an “Other” category for further analyses because they had very small numbers. In comparison, the main diagnoses at the hospitals were malaria (98), anaemia (62), pneumonia (11) and diarrhoea (7). The category “No Diagnosis” was made for 24 patients because no further information could be traced upon revisits to the hospital. “Abdominal Burkitt's Lymphoma”, “Asthma” “Clavicular Fracture”, “Congenital Heart Disease”, “Convulsion”, “Gastroenteritis”, “Hepatitis”, “Intestinal Parasites”, “Sepsis” and “Skin Infection” were grouped in “Other” category for further analyses because numbers were small.

Table 2 presents a comparison of diagnoses at a district hospital with diagnosis for the same patient at a PHC. PHC centres diagnosed a total of 4 patients with “Abdominal” condition; of the 4 patients, only one (25%) was diagnosed as having diarrhoea with remaining 3(75%) diagnosed as having “Malaria”. There were 68 patients diagnosed for “Anaemia” at PHC centres but in the hospitals 47(69%) were diagnosed as having “Anaemia”, with remaining diagnosed as “Malaria” 20(29%) and Pneumonia 1(2%).

**Table 2:** Diagnoses made at the PHC Centre compared with those made at the hospital

Hospital Diagnosis	ABDOMINAL	ANAEMIA	CONVULSION	DIARRHEOA	FEVER	MALARIA	VOMITING	Total
ANAEMIA	0 (0)	47 (69.1)	1 (10)	0 (0)	6 (17.1)	7 (16.3)	0 (0)	**61**
DIARRHOEA	3 (75)	0 (0)	0 (0)	1 (14.3)	0 (0)	1 (2.3)	3 (75)	**6**
MALARIA	1 (25)	20 (29.4)	9 (90)	4 (57.1)	23 (65.7)	34 (79.0)	1 (25)	**94**
PNEUMONIA	0 (0)	1 (1.5)	0 (0)	2 (28.8)	6 (17.1)	1 (2.3)	0 (0)	**10**
Total	4 (100)	68 (100)	10 (25)	7 (100)	35 (100)	43 (100)	4 (100)	**171**

Note: The Chi-Square Test of Independence between hospital diagnosis and health centre diagnosis was applied to the contingency table (Table 2) and it showed that there is an association between the diagnosis made by the hospital and that made by the health centre, Chi2(18) = 141, Fisher's exact p < 0.001.

There were 10 Convulsion cases diagnosed at PHC centres compared with hospital diagnoses of 1 (10%) case of Anaemia and 9 (90%) cases of Malaria, and no convulsion. PHC centres diagnosed seven patients for Diarrhoea as against 1(14%) case of Diarrhoea, 4 (57%) cases of Malaria and 2(29%) cases Pneumonia for same patients when they visited the hospitals. Fever cases diagnosed at PHC centres were in 35 patients as against no fevers in the hospital but instead 23(66%) of the patients were diagnosed as having Malaria, 6 (17%) cases with Anaemia and 6(17%) of Pneumonia. PHC centres diagnosed 50 patients for Malaria compared with 34 (68%) patients with Malaria, 7(17%) with Anaemia, 1(2%) with Diarrhoea and 1 (2%) with Pneumonia in the hospital. Vomiting was diagnosed in 4 patients by PHC centres as against 3 (75%) patients with Diarrhoea and 1 (25%) with Malaria at the hospital.

As high as 69% of anaemia cases diagnosed at PHC Centres were diagnosed at the hospital but only 29% of anaemia cases diagnosed at the PHC centres were associated with malaria at hospital level. Most fever cases (65.7%) diagnosed at PHC Centre level had confirmatory diagnosis of malaria at the hospital level; the respective agreement between PHC centre and hospital diagnosis for fever-anaemia and fever-pneumonia were only 17% each.

The level of agreement between malaria diagnosis at PHC centre and hospital was 78%; only 29.4% of malaria cases diagnosed at the hospital were reported to have anaemia in PHC centres Table 2.

### Health care providers' perspectives on adherence to referrals by carers

Most healthcare providers referred children to ensure they benefited improved care that did not exist in their PHCs. Specific reasons for referral included severity and complication, non-response to treatment (high persistent temperature, low levels of haemoglobin) and carers loss of trust in the care available at PHC level.

PHC providers perspectives of what motivates carers of children to accept referrals, included: child's worsening condition requiring referral.

*“I said earlier that if you explain to carer this is the nature of your child's condition and you tell the carer this is what will happen to your child if you don't go to the hospital, the person will accept and go. If you do not explain and you give just the letter to the person, he/she may decide not to go” (In-charge, PHC)*

Some carers refused referrals, because they lack understanding why they should comply with referral advice and others due to financial constraints.

*“Refusal is as a result of the lack of knowledge, so if our education can go on well, I think the refusals can be minimized. Some of them too don't have money for transportation and it usually come with comments such as “mi kunu nni hô” [my husband is not there]. With these excuses, they will always say I will go home to look for money so they go home and remain there” (PHC Centre In-charge)*

Long queues in district hospitals and fear of going to die were other factors.

*“Some of them say it is the long queue; they don't want to go and queue there for a long time but I always explain to them that that is the main referral facility in our area so they will have to go there” (PHC Physician Assistant).*

Healthcare providers reported of some patients dying as a result of non-adherence to referral.

*“The first child was anaemic and we referred the child. After the referral we couldn't follow them again because the child is from the other community.*

*They didn't send the child to hospital. We later found out that the child has died and when we confronted them, the mother said the husband was not around and there was no money for transportation” (In-charge, PHC).*

Though required to feedback, healthcare providers at referral facilities blamed their inability to feedback on heavy workload at the hospital.

*“Unfortunately for us we should have been getting feedback from the hospital, but they are also complaining about high work load……” (In-Charge, PHC).*

Healthcare providers referring patients are to check on status of their patients.

*“Some of them if we have their telephone numbers we call them to find out how they are doing” (In-charge, PHC)*

Respondents mentioned some carers who do not adhere to referral seek care from traditional healers and herbalists.

*“Some go there, or some even go there before coming here; that one too was one child; convulsion they sent the person there and when they failed they brought the child here and I realized it was anaemia so I referred the child” (In-charge, PHC)*

## Discussion

Though millions of carers of children in Ghana seek healthcare every year^19^, there still remain numerous inherent obstacles to children receiving timely, quality care.^19^–^21^ These study findings provide information on referral system and adherence to referral in Ghana, highlighting some solutions.

Though most children in this study were managed at 32 PHC centres, it is of interest that only 7% of the patients were referred. Though there was concern, only few deaths 1.1% (34) occurred throughout the follow-up period. It can be inferred that children largely received appropriate medical care at the PHCs.

It is possible that there were some children among the 2834 who should have been referred but were not; pointing to some of the health systems deficiencies such as delays in health workers responses to some of the conditions of participants, attitude of some health workers, inadequate qualified personnel, limited supplies as well as missed opportunities for testing which leads to delayed diagnosis. Though referral rate in this study is relatively low, findings from another study in Burkina Faso revealed much lower referral rate of 2%(of 45,356 patients) in 8 rural PHC centres, were referred to a higher level facility.^22^ That notwithstanding, referral rate in this study is lower compared to other studies carried out in Sierra Leone, with 15% referral rate reported in a communitybased project where Community Malaria Volunteers tested and treated febrile children and pregnant women for malaria using Rapid Diagnostic Tests whilst RDT negative and severely ill patients were referred to health facilities.^23^ Assessment of severity of patients' condition requiring referral by healthcare providers should be addressed through a strengthened peer-education programme.

Adherence levels among referred patients was high (86%) in this study; and comparable to 86.6%, 83% and 76% adherence levels in the Netherlands^24^, USA^25^, and Afghanistan^5^ respectively, when patients were referred from PHC centres to district hospitals. Adherence level in this study is, however, higher than that reported from other African countries. In Niger, 55% of patients presented with emergency conditions adhered to referrals. 26 Findings from Burkina Faso documented 41.5% 22 and Nigeria 37.8%. 11 Some reasons that could be attributed to the high adherence to patient's referral in our study include close to half (48.3%) of carers who were referred perceived that the health condition of their children were severe enough to warrant referral; Also 43.4% of carers trusted the health workers judgement/clinicians diagnosis about their children when they were referred. These findings are consistent with findings from Guatemala where severity of the illness was considered as most compelling reason for compliance to referral.^4^

Some of the reasons for the high level of adherence we observed was the fact that our field teams stayed in the same communities as the study participants and visited them every month. We removed financial barrier to accessibility by registering all study participants on the National Health Insurance Scheme. Carers/parents therefore did not need to pay to access services.

Some carers may have gone to the hospital themselves, because they may have been dis-satisfied with diagnosis and/or treatment they had at PHC centre in the past. 27, 28 This finding is contrary to general principle of patient referral where referral networks are intended to move clients up through a pyramid-shaped structure starting from PHC to higher levels of care at district, regional or national hospital, or private facility due to severity of illness and availability of service.^29^

Inability of carers adherence to referrals of their children could put the child's life in danger. 30 Information about what hinders carers from adhering to referrals was therefore collected/collated, to help in developing interventions that could prevent detrimental outcomes of sickchildren and improve the referral systems in our communities.

Transportation costs emerged as one of the major reasons why carers did not adhere to referrals as 50% of defaulters attributed their inability to transportation.^24^ Beyond the direct cost of treatment, which is borne by the National Health Insurance Authority for children insured, the cost of transportation and upkeep, care for other siblings of referred child and accommodation while out for the referral are borne directly by caregivers household. The carers who did not adhere to referral were, however, in terms of their socio-economic status were not significantly different from those who adhered. The profile of parents/carers of the 30 children who did not adhere compared to the 182 children who adhered was [Pearson chi^2^(4) = 6.2283, p-value= 0.183].In non-emergency situations, the carers may think the child is well enough and need not go to hospital. They may depend on initial medications provided or instead go to a nearby license chemist shop to buy some medication for the child 31

Another challenge carers encounter are long queues in the referral facilities due to high numbers of patients seeking care at few available hospitals. With several PHC facilities that could be visited in the study area, there were only four fully functional district hospitals; one hospital serves as a major referral facility for all the catchment PHC facilities in the District.

Lack of knowledge of the need to comply with referral as a challenge is consistent with demographic trends at the study site. 32 This is because per the socio-cultural practices in the study site, most carers are likely to be females with low-levels of education. Data for females in the study area have 55.2% with no education, 23.2% with primary education and 3.7% with secondary education. 32 Another reason that could have contributed to lack of compliance is health workers not explaining the severity of the illness to the parents appropriately. There is also a strong belief/perception that affects adherence; though hospital is the place for treatment of diseases, some community members belief it is also the place to die.^33^–^35^

There was a high level of agreement in diagnosis made for common ailments such as malaria, anaemia and diarrhoea at PHC centres compared with hospitals. This is a clear demonstration of adequacies in managing simple ailments at PHC. It was, however, not the case for prescribers at PHC centres when it comes to diagnosing ailments like pneumonia. Anaemia and malaria are the commonest diseases recorded at most Outpatient Departments (OPD) Health facilities in Ghana^9^,^36^,^37^ as they can easily be diagnosed using simple equipment like automated haemocues and malaria rapid diagnostics tests. This finding is in agreement with technical assessment perspective of measuring quality of care, which provides evidence-based criteria that can be associated with health outcomes.^38^ Results from a cross-sectional survey in Zimbabwe evaluating the quality of health care within first line health services did not provide conclusive evidence that the care offered at PHC centre was better or worse than care available at hospital OPD.^39^ Carers can be assured on aspects of our findings including high quality care at PHC levels and patronise services closest to them.

It became evident from our interactions with healthcare providers, that the National Health Insurance Scheme (NHIS) introduced some barriers in appropriate management of children; a patient with malaria cannot be treated more than once in the same month. Such cases are referred by the healthcare provider at PHC centre to the district hospital.

Though the structures for feedback exist within the Ghana health care delivery system, this was not adhered to. It was observed in this study that health workers in the higher referral facilities did not complete required forms that help provide feedback to those in lower facilities on status of referred patients. This is contrary to the PHC concept where referral serves as a linkage between primary and secondary health care.^40^

One limitation in our findings is that, we did not collect data to inform us on the form of communication between health providers and caregivers at the health centre and hospital levels.

## Conclusion

Adherence to referrals made from PHC centres to district hospitals is quite high and can be further improved through targeted approaches. Availability of transport to convey patients from home to health facilities and back as well as ability of parents/carers to pay for transportation to the health facility needs to be explored further. There is also the need to continue to build on the trust patients and carers of patients have in the healthcare workers as such trust will impact on adherence to referrals. There was a high level of agreement in the diagnosis of common ailments such as malaria and anaemia at PHC centres and district hospitals, but not for others like pneumonia. Enhanced training for clinicians at PHCs and improved availability of diagnostics tools for common diseases could greatly improve and rationalise the referral system.

## Figures and Tables

**Table 1:** Tabulation of Diagnoses/symptoms made at the health Centre against those made at the hospital

	Hospital diagnosis														
HC diagnosis	ABDO	ANAEMIA	ASTHMA	CONGENIT	CONVUL	DIAR	GASTROE	HEPATIT	INTESTIN	MALARIA	NO DIAG	PNEUMONIA	SEPSIS	SKIN INFEC	Total
**ABDOMINAL**	1	0	0	0	0	1	0	0	0	3	0	0	0	0	5
**ANAEMIA**	0	47	0	0	0	0	0	0	0	20	0	1	0	0	68
**ARTI**	0	0	0	0	0	0	0	0	0	0	1	0	0	0	1
**ASTHMA**	0	0	1	1	0	0	0	0	0	0	0	0	0	0	2
**CONSTIPATION**	0	0	0	0	0	0	0	0	0	0	1	0	0	0	1
**CONVULSION**	0	1	0	0	0	0	0	0	0	9	3	0	0	0	13
**COUGH**	0	1	0	0	0	0	0	0	0	0	0	0	0	0	1
**DIARRHOEA**	0	0	0	0	0	1	0	0	0	4	1	2	0	0	8
**DISLOCATION**	0	0	0	0	0	0	1	0	0	0	0	0	0	0	1
**FEVER**	0	6	0	0	0	0	0	1	0	23	9	6	0	0	45
**MALARIA**	0	7	0	0	1	1	0	0	0	34	4	1	2	0	50
**ORAL THRUSH**	0	0	0	0	0	0	0	0	1	0	0	0	0	0	1
**RESTLESSNESS**	0	0	0	0	0	1	0	0	0	0	0	0	0	0	1
**SKIN DISEASE**	0	0	0	0	0	0	0	0	0	0	1	0	0	0	1
**SKIN INFECTION**	0	0	0	0	0	0	0	0	0	0	1	0	0	1	2
**TYPHOID**	0	0	0	0	0	0	0	0	0	1	0	0	0	0	1
**URTI**	0	0	0	0	0	0	0	0	0	1	0	0	0	0	1
**VOMITING**	0	0	0	0	0	3	0	0	0	1	2	0	0	0	6
**WEAKNESS**	0	0	0	0	0	0	0	0	0	2	0	0	0	0	2
**YELLOW COLOR**	0	0	0	0	0	0	0	0	0	0	1	0	0	0	1
**YELLOW FEVER**	0	0	0	0	0	0	0	0	0	0	0	1	0	0	1
**Total**	**1**	**62**	**1**	**1**	**1**	**7**	**1**	**1**	**1**	**98**	**24**	**11**	**2**	**1**	**212**

Note: ABDO = abdominal pain; CONGENIT = congenital heart disease; CONVUL= convulsion; DIAR= diarrhoea; GASTROE = gastroenteritis; INSTESTIN = intestinal parasites; NO DIAG = no diagnosis; SKIN INFEC = skin infection
